# Non-invasive prediction of preeclampsia using the maternal plasma cell-free DNA profile and clinical risk factors

**DOI:** 10.3389/fmed.2024.1254467

**Published:** 2024-04-17

**Authors:** Yan Yu, Wenqiu Xu, Sufen Zhang, Suihua Feng, Feng Feng, Junshang Dai, Xiao Zhang, Peirun Tian, Shunyao Wang, Zhiguang Zhao, Wenrui Zhao, Liping Guan, Zhixu Qiu, Jianguo Zhang, Huanhuan Peng, Jiawei Lin, Qun Zhang, Weiping Chen, Huahua Li, Qiang Zhao, Gefei Xiao, Zhongzhe Li, Shihao Zhou, Can Peng, Zhen Xu, Jingjing Zhang, Rui Zhang, Xiaohong He, Hua Li, Jia Li, Xiaohong Ruan, Lijian Zhao, Jun He

**Affiliations:** ^1^Department of Obstetrics, Shenzhen Baoan Women’s and Children’s Hospital, Shenzhen, China; ^2^BGI Genomics, BGI-Shenzhen, Shenzhen, China; ^3^Hebei Industrial Technology Research Institute of Genomics in Maternal and Child Health, Shijiazhuang BGI Genomics, Shijiazhuang, Hebei, China; ^4^Department of Clinical Laboratory (Institute of Medical Genetics), Zhuhai Center for Maternal and Child Health Care, Zhuhai, China; ^5^Department of Obstetrics and Gynecology, Jiangmen Central Hospital, Jiangmen, Guangdong, China; ^6^BGI-Tianjin, BGI-Shenzhen, Tianjin, China; ^7^The First Affiliated Hospital, Sun Yat-Sen University, Guangzhou, China; ^8^Department of Prevention and Health Care, Zhuhai Center for Maternal and Child Health Care, Zhuhai, China; ^9^Department of Genetics and Eugenics, Changsha Hospital for Maternal and Child Health Care, Changsha, China; ^10^Hunan Provincial Key Laboratory of Regional Hereditary Birth Defects Prevention and Control, Changsha Hospital for Maternal and Child Health Care Affiliated to Hunan Normal University, Changsha, China; ^11^Hospital Office, Changsha Hospital for Maternal and Child Health Care, Changsha, China; ^12^Department of Medical Genetics and Prenatal Diagnosis, Baoan Women’s and Children’s Hospital, Jinan University, Shenzhen, China; ^13^Hebei Medical University, Shijiazhuang, Hebei, China

**Keywords:** preeclampsia, non-invasive prenatal testing, cell-free DNA, prediction, *in vitro* fertilization

## Abstract

**Background:**

Preeclampsia (PE) is a pregnancy complication defined by new onset hypertension and proteinuria or other maternal organ damage after 20 weeks of gestation. Although non-invasive prenatal testing (NIPT) has been widely used to detect fetal chromosomal abnormalities during pregnancy, its performance in combination with maternal risk factors to screen for PE has not been extensively validated. Our aim was to develop and validate classifiers that predict early- or late-onset PE using the maternal plasma cell-free DNA (cfDNA) profile and clinical risk factors.

**Methods:**

We retrospectively collected and analyzed NIPT data of 2,727 pregnant women aged 24–45 years from four hospitals in China, which had previously been used to screen for fetal aneuploidy at 12 + 0 ~ 22 + 6 weeks of gestation. According to the diagnostic criteria for PE and the time of diagnosis (34 weeks of gestation), a total of 143 early-, 580 late-onset PE samples and 2,004 healthy controls were included. The wilcoxon rank sum test was used to identify the cfDNA profile for PE prediction. The Fisher’s exact test and Mann–Whitney U-test were used to compare categorical and continuous variables of clinical risk factors between PE samples and healthy controls, respectively. Machine learning methods were performed to develop and validate PE classifiers based on the cfDNA profile and clinical risk factors.

**Results:**

By using NIPT data to analyze cfDNA coverages in promoter regions, we found the cfDNA profile, which was differential cfDNA coverages in gene promoter regions between PE and healthy controls, could be used to predict early- and late-onset PE. Maternal age, body mass index, parity, past medical histories and method of conception were significantly differential between PE and healthy pregnant women. With a false positive rate of 10%, the classifiers based on the combination of the cfDNA profile and clinical risk factors predicted early- and late-onset PE in four datasets with an average accuracy of 89 and 80% and an average sensitivity of 63 and 48%, respectively.

**Conclusion:**

Incorporating cfDNA profiles in classifiers might reduce performance variations in PE models based only on clinical risk factors, potentially expanding the application of NIPT in PE screening in the future.

## 1 Introduction

Preeclampsia (PE) is a pregnancy complication defined by new onset hypertension and proteinuria or other maternal organ damage (e.g., kidney, liver or brain) after 20 weeks of gestation (1–4). The overall prevalence of PE is about 2–8% (2, 5). PE and its related complications of hypertensive disorders are the second leading cause of maternal mortality (6, 7). Depending on the time of diagnosis (34 weeks of gestation), PE is divided into early- and late-onset PE (8–10). As the disease progresses, the main treatment for PE remains the termination of pregnancy. However, premature delivery can lead to adverse consequences for the fetus. Previously clinical trials showed using low-dose aspirin in early pregnancy may reduce the risk of PE in pregnant women at high risk (11). Therefore, there is a need for an early and accurate PE prediction method, especially for early-onset PE.

To date, the history of PE, chronic hypertension and diabetes mellitus, older age, higher body mass index (BMI) and *in vitro* fertilization (IVF) are known to increase the risk of PE in pregnant women (2, 12, 13). However, models based on these clinical risk factors alone usually showed low accuracy or high false positives (14, 15). The Fetal Medicine Foundation (FMF) has proposed a combined predictive model incorporating mean arterial pressure (MAP), uterine artery pulsatility index (UtA-PI), serum placental growth factor (PLGF) and pregnancy-associated plasma protein-A (PAPPA) for the evaluation of PE risk in the first trimester (2, 16–19). With a false positive rate (FPR) of 10%, the detection rates were 60.7 and 21.3% for preterm and term PE in mainland China, respectively. The demanding and complex measurement technique of UtA-PI makes the method difficult to be widely used, so a more general and convenient method is needed to predict pregnant women at high risk of PE.

Cell-free DNA (cfDNA) was first discovered in 1948 (20). It is mainly derived from apoptotic cells in healthy or diseased individuals. In pregnant women, about 10–15% of cfDNA originates from placental trophoblasts (21). Transcriptionally active gene promoters are nucleosome-depleted regions in the placenta. Naked DNA fragments are more susceptible to degradation as they enter the circulatory system. Lower levels of cfDNA derived from these promoters are exhibited. Thus, the abundance of cfDNA in maternal plasma could reflect gene expression in the placenta. Abnormal gene expression in the placenta might cause placental dysfunction. Therefore, cfDNA in the plasma of pregnant women might have the potential to be used as a biomarker for the diagnosis and prediction of diseases caused by placental dysfunction during pregnancy. Recently, studies have determined the promoter profiling, the concentration and DNA methylation of maternal plasma cfDNA can be used to predict pregnancy complications (22–28). However, it remains unknown whether the non-invasive prenatal testing (NIPT) data used to screen for fetal chromosomal abnormalities could also be used for PE prediction.

Therefore, we aimed to uncover cfDNA biomarkers with predictive value for PE by analyzing NIPT data. We evaluated whether the predictive performances of PE classifiers based on cfDNA coverages of gene promoter regions and clinical risk factors could be validated in internal and external validation datasets and showed reasonable accuracies, which might extend the use of NIPT for PE screening in the future.

## 2 Materials and methods

### 2.1 Participants

In the study, we retrospectively collected NIPT data of 2,727 pregnant women aged 24–45 years who underwent NIPT at 12 + 0 ~ 22 + 6 weeks of gestation at four hospitals in China from 2019 to 2021, including Zhuhai Center for Maternal and Child Health Care, Shenzhen Baoan Women’s and Children’s Hospital, Changsha Hospital for Maternal and Child Health Care and Jiangmen Central Hospital. This study was approved by the Ethics Committees of Beijing Genomics Institute (BGI) and these four hospitals (BGI-IRB 22026, LLSC-2022-01-04-04-KS, 2022030 and [2022]02), and all the participants provided their informed consent at the time of NIPT.

PE patients were diagnosed according to the following criteria: maternal systolic blood pressure is ≥140 mmHg and/or diastolic blood pressure is ≥90 mmHg after 20 weeks of gestation with the proteinuria level of ≥0.3 g/24 h or dipstick urine test ≥1+. Early- and late-onset PE were classified based on the time of PE diagnosis (34 weeks of gestation) (8–10). Pregnant women with PE were excluded if they had malignant neoplastic diseases or fetal chromosomal abnormalities. Pregnant women with singleton pregnancy were considered as healthy controls if they did not meet the following criteria: (1) pregnancy complications, such as PE, gestational diabetes mellitus and cholestasis; (2) severe cardiac, hepatic and renal insufficiency; (3) malignant neoplastic diseases; (4) fetal chromosomal abnormalities. Moreover, PE and healthy individuals who had taken aspirin before sampling were excluded from the study cohort. A total of 143 early-onset PE samples, 580 late-onset PE samples and 2,004 healthy controls with complete clinical information were included in this study.

### 2.2 cfDNA extraction, library preparation and sequencing

Five milliliters of maternal peripheral blood were collected into Streck Cell Free DNA BCT® blood collection tubes (Streck, La Vista, NE, United States). Two hundred microliters of maternal plasma were used for cfDNA extraction, library construction and sequencing as described previously (29). Briefly, end repairing and adding a non-template dA tail to cfDNA were carried out and DNA amplification products were obtained by polymerase chain reaction (PCR). Then, the amplified double-stranded DNA was thermally denatured to single-strand DNA, cyclized and made into DNA nanoballs (DNBs). Finally, the DNBs were loaded onto chips and sequenced on the BGISEQ-500 sequencing platform (BGI, Shenzhen, China). The average depth of coverage is 0.1X.

### 2.3 Low-coverage whole-genome sequencing data processing

Raw reads were aligned to the hg38 human reference using BWA aln (30). After extracting uniquely mapped reads based on Sequence Alignment/Map (SAM) tags, removing PCR duplicates was performed using SAMTOOLS (31).

### 2.4 Analysis of the cfDNA profile

To identify the cfDNA profile in NIPT data that could stably predict pregnant women at high risk of early- or late-onset PE in different hospitals, we analyzed maternal plasma cfDNA coverages at primary transcription start sites (pTSSs), called pTSS coverage (Supplementary Figure 1A). For each transcript downloaded from GENCODE (v37, 32), we used the pTSS as a promoter region, which is a 2 kb region centered on the transcription start site. Read coverages at pTSSs were calculated from aligned BAM files using BEDtools (ver. 2.29.2, 33) and normalized using the reads per kilobase per million mapped reads (RPKM) method. Then, we calculated the multiple of the median (MoM) value of each pTSS coverage by dividing the raw value by the median value of control samples in each dataset.


$${\mathrm{pTSS}}_{i}\;\mathrm{coverage}=\frac{\mathrm{The}\;\mathrm{number}\;\mathrm{of}\;\mathrm{mapped}\;\mathrm{reads}\;\mathrm{on}\;\mathrm{the}\;{TSS}_{i}\times 10^{9}}{\mathrm{The}\;\mathrm{total}\;\mathrm{number}\;\mathrm{of}\;\mathrm{mapped}\;\mathrm{reads}\times 2000}$$

### 2.5 Pathway enrichment analysis

The Kyoto Encyclopedia of Genes and Genomes (KEGG) and WikiPathways analyses were performed using g: Profiler (34). The *p* < 0.05 by computing multiple testing correction was considered statistically significant.

### 2.6 Experimental design

**Figure 1:**
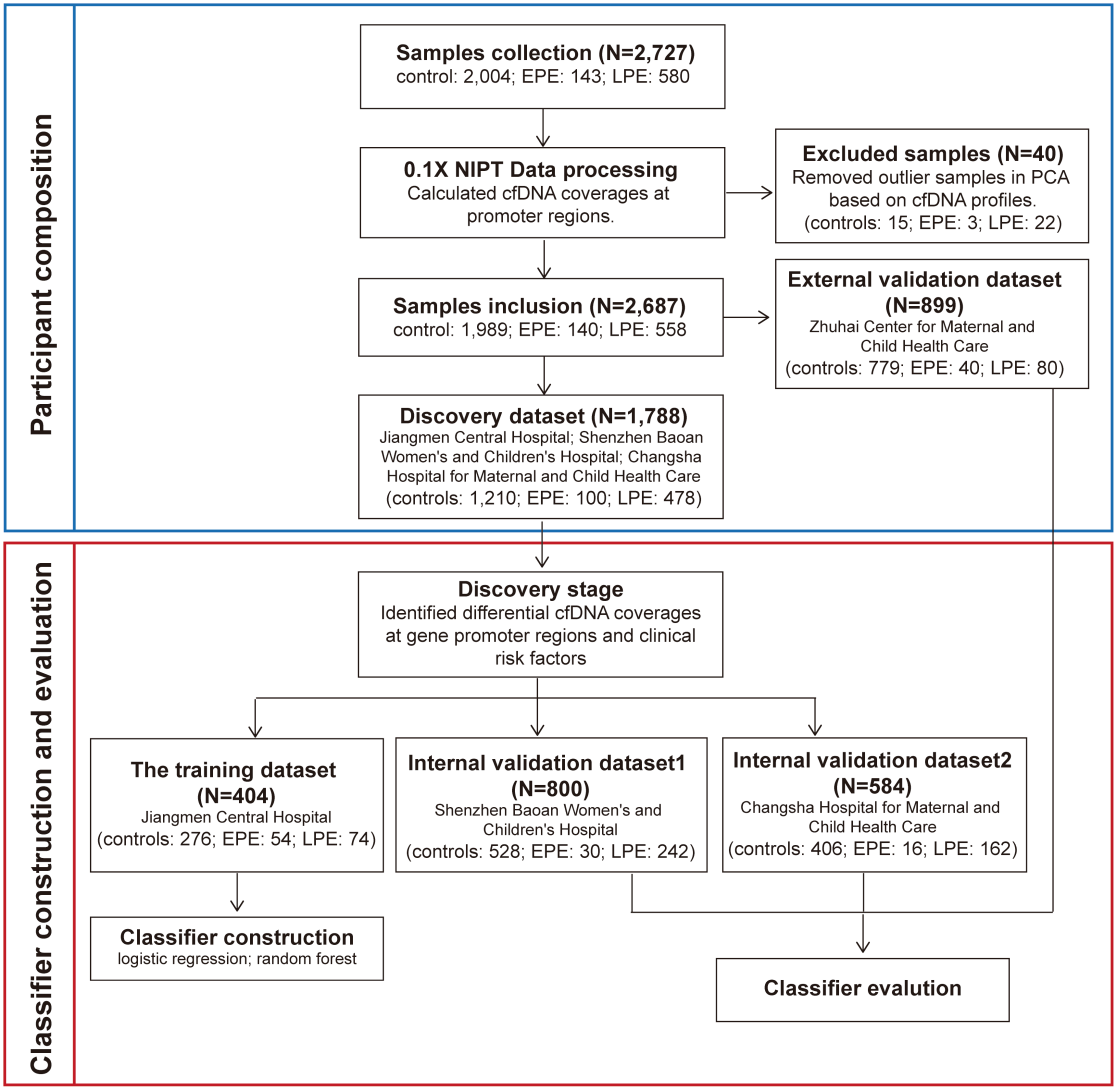
The workflow for developing early- and late-onset PE classifiers. We collected 2,727 NIPT data from pregnant women at 12 + 0 ~ 22 + 6 weeks of gestation from four hospitals. The cfDNA profiles which were cfDNA coverages at gene promoter regions were identified. Before the construction and evaluation of classifiers for PE prediction, 40 samples were removed based on the PCA analysis. The remaining 2,687 NIPT data were used as the training and three validation datasets. Samples from the same hospital were assigned to a dataset. In the discovery stage, clinical risk factors and the cfDNA profile used to develop PE classifiers were screened. In the classifiers construction stage, samples from Jiangmen Center Hospital were used to build classifiers by the machine learning method. In the evaluation stage, the classifiers were further validated in three validation datasets.

We followed the workflow encompassing sample allocation, classifier construction, and classifier evaluation. We first calculated pTSS coverages of 2,727 NIPT data as described above and performed the principal components analysis (PCA) using pTSS coverages. Samples whose the mean values of the first and second principal components differed within three standard deviations were retained. The PCA excluded 3 early-onset PE, 22 late-onset PE and 15 control samples (Figure 1; Supplementary Figure S1B). Then, we chose 899 samples from the Zhuhai Center for Maternal and Child Health Care as an external validation set. The remaining 1,788 NIPT data were used as the discovery dataset to identify cfDNA coverages at gene promoters regions and clinical risk factors relevant to PE prediction. In the discovery stage, the differential analysis of pTSS coverages was performed 1,000 times, each time using 70% samples randomly sampled from the discovery dataset. The wilcoxon rank sum test was used to identify significantly differential pTSS coverages between PE samples and healthy controls. The pTSS coverage was considered as a candidate cfDNA biomarker if the *p*-value (the upper limit of 95% CI for 1,000 times) < 0.05 and had the same trend of change. For clinical characteristics, MoM values of maternal age and BMI were calculated. The parity, past medical history and method of conception were discrete values. The past medical history was the sum of number of histories of chronic hypertension, PE, systemic lupus erythematosus (SLE) and antiphospholipid syndrome, on a scale of 0–4. The method of conception was defined as 1 for IVF and 0 for natural pregnancy. To retain the independence of different hospitals, the samples from the same hospital were assigned to a single dataset. In the classifier construction stage, machine learning methods (logistic regression, LR and random forest, RF) were performed to construct PE classifiers using samples from Jiangmen Central Hospital. Receiver operating characteristic (ROC) analysis was used to evaluate the performance of each classifier. In the evaluation stage, classifiers were validated in two internal validation datasets (800 samples from Shenzhen Baoan Women’s and Children’s Hospital, 584 samples from Changsha Hospital for Maternal and Child Health Care) and an external validation dataset (899 samples from Zhuhai Center for Maternal and Child Health Care). The 95% confidence intervals of area under curves (AUCs) of the training set and validation sets were calculated from 1,000 bootstrap samples. The sample size of each random sampling was equal to the size of the original dataset. ROC curves were plotted and AUC, sensitivity and specificity values were calculated by custom scripts in python to evaluate performances of classifiers.

### 2.7 Statistical analyses

The Fisher’s exact test was used to compare categorical variables between PE samples and healthy controls, such as gravidity, parity, past medical history and method of conception. The Mann–Whitney U-test was used to compare continuous variables, such as maternal age and BMI. The DeLong’s test was used to compare AUCs. *p* < 0.05 was considered statistically significant.

## 3 Results

### 3.1 Clinical characteristics for study cohorts

We retrospectively collected NIPT data of pregnant women at 12 + 0 ~ 22 + 6 weeks of gestation from 2019 to 2021 at four hospitals in China. A total of 2,727 individuals with complete clinical information met the enrollment criteria (Figure 1). This study included four datasets: a training dataset, two internal validation datasets and one external dataset. The demographic and pregnancy details of pregnant women in the training and validation datasets were shown in Table 1 and Supplementary Table S1. As expected, pregnant women with older maternal age, higher BMI, past medical histories (chronic hypertension, PE, SLE or antiphospholipid syndrome) or IVF were more likely to develop early- or late-onset PE in at least two datasets. By contrast, parous women had a decreased risk of developing LPE in three datasets (Table 1; Supplementary Table S1). The maternal age, BMI, past medical histories, parity and IVF were subsequently used to construct classifiers for predicting early- and late-onset PE.

**Table 1:** Demographic and clinical characteristics of early-onset PE patients and healthy controls in the training and validation datasets.

	Training set		Internal validation dataset1		Internal validation dataset2		External validation dataset	
	Control	EPE	Control	EPE	Control	EPE	Control	EPE
Number of subjects	276	54	528	30	406	16	779	40
Maternal age (years)	28.0 (26.0–31.0)	33.0 (30.0–36.0)***	29.0 (27.0–31.0)	31.0 (28.3–34.0)**	31.0 (29.0–33.0)	32.0 (31.0–34.3)	29.0 (27.0–32.0)	31.0 (29.0–35.0)***
Height (cm)	160.0 (156.0–163.0)	160.0 (155.0–165.8)	160.0 (156.0–163.0)	160.0 (156.0–161.0)	161.0 (158.0–165.0)	161.5 (157.8–164.3)	160.0 (157.0–164.0)	158.0 (155.0–162.0)*
Weight (kg)	52.5 (47.0–57.0)	57.0 (51.0–63.0)***	53.0 (48.0–58.0)	56.7 (51.6–60.4)*	54.5 (50.0–59.0)	59.5 (55.5–68.0)**	54.0 (50.0–60.0)	58.0 (53.0–67.3)***
BMI (kg/m^2^)	20.5 (18.8–22.5)	22.2 (20.3–23.9)***	20.6 (19.1–22.7)	21.8 (20.2–24.2)*	20.8 (19.6–22.4)	22.9 (21.4–24.8)**	21.1 (19.5–22.9)	24.0 (21.4–27.6)***
GA at delivery (weeks)	39.4 (38.6–40.1)	33.1 (31.0–34.3)***	39.0 (38.0–40.0)	32.4 (30.0–33.6)***	39.0 (39.0–40.0)	33.5 (31.1–34.0)***	39.0 (38.0–40.0)	32.4 (29.0–35.0)***
Gravidity ≥1, *n* (%)	276 (100.0)	54 (100.0)	528 (100.0)	30 (100.0)	406 (100.0)	16 (100.0)	779 (100.0)	40 (100.0)
Parity ≥1, *n* (%)	157 (56.9)	25 (46.3)	298 (56.4)	11 (36.7)	164 (40.4)	7 (43.8)	279 (35.8)	20 (50.0)
PMH ≥1, *n* (%)	0 (0.0)	3 (5.6)**	0 (0.0)	1 (3.3)	0 (0.0)	1 (6.3)*	0 (0.0)	5 (12.5)***
IVF, *n* (%)	5 (1.8)	14 (25.9)***	0 (0.0)	7 (23.3)***	0 (0.0)	0 (0.0)	15 (1.9)	3 (7.5)

Data were shown as median values (interquartile range) of clinical characteristics or numbers (percentages) of samples. *, ** and *** represented the different significance of *p* < 0.05, *p* < 0.01 and *p* < 0.001 between early-onset PE and control samples, respectively. EPE, early-onset preeclampsia; BMI, body mass index; PMH, past medical history; GA, gestational age; IVF, *in vitro* fertilization.

### 3.2 Characterization of differential cfDNA profiles between PE and control samples

**Figure 2:**
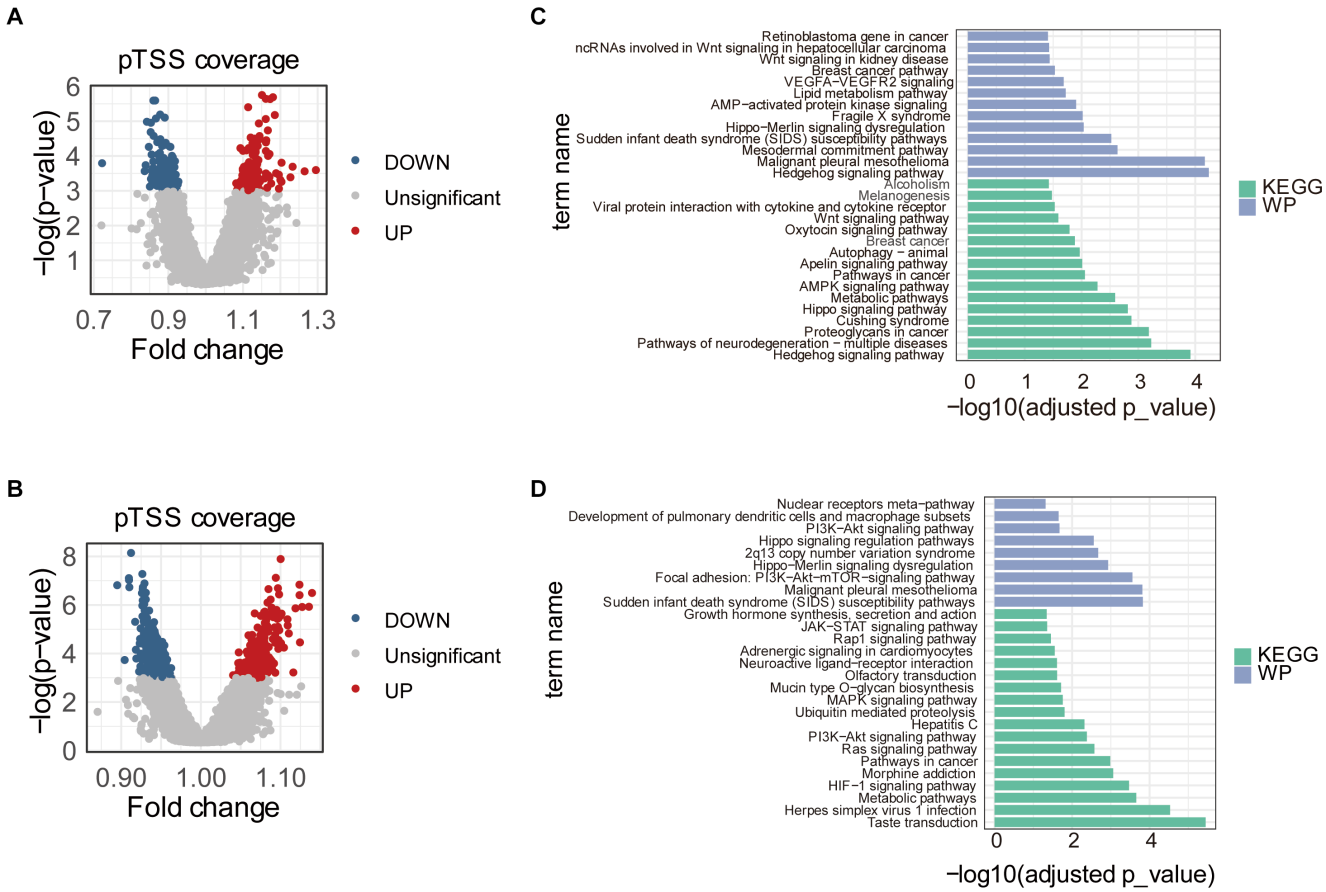
Differential pTSS coverages between early- or late-onset PE and controls. Volcano plots showed promoters with differential cfDNA coverages between early-onset PE and healthy samples **(A)** or between late-onset PE and healthy samples **(B)**. The red, blue, and grey dots indicated pTSS coverages were significantly higher, lower, and non-significant in early- or late-onset PE compared to control samples, respectively. Barplots showed the results of pathway enrichment analysis based on genes with differential pTSS coverages between early-onset PE and healthy samples **(C)** or between late-onset PE and healthy samples **(D)**. KEGG, Kyoto Encyclopedia of Genes and Genomes; WP, WikiPathways.

To find promoters with stably differential cfDNA coverages between PE and control samples, we repeated the differential analysis 1,000 times. Each time, 70% of the samples in training and internal validation datasets were randomly sampled and used. In total, pTSS coverages of 117 and 137 promoters were significantly higher and lower in early-onset PE samples compared to healthy controls, respectively (Figure 2A). Similarly, there were 266 and 344 promoters with significantly differential higher and lower pTSS coverages in late-onset PE samples compared to healthy controls (Figure 2B). Of these, pTSS coverages of *CACNB2* and *NRF1* gene promoters were lower in both early- and late-onset PE samples.

Then, we performed the KEGG pathway and WikiPathways enrichment analyses using genes with significantly differential cfDNA coverages in promoters between early- or late-onset PE and control samples. The results of pathway analyses showed that these genes were enriched in multiple pathways associated with PE (Figures 2C,D). For early-onset PE, genes with significantly differential cfDNA coverages in promoters enriched in “Hedgehog signaling pathway,” “Hippo signaling pathway,” “AMPK signaling pathway,” “Apelin signaling pathway,” “Autophagy,” “Oxytocin signaling pathway,” “Wnt signaling pathway” and “VEGFA-VEGFR2 signaling.” For late-onset PE, the results of pathways enriched in “HIF-1 signaling pathway,” “Ras signaling pathway,” “PI3K-Akt signaling pathway” and “MAPK signaling pathway”.

### 3.3 Development and validation of classifiers based on the cfDNA profile and clinical risk factors

For early-onset PE prediction, the cfDNA profile and clinical risk factors were combined to construct the early-onset PE classifier (C_EPE_). The cfDNA profile included pTSS coverages of *FOSL2*, *CAMKK2*, *CCND1*, *ITPR1*, *PRKACB* and *WNT7B* genes, which played roles in least three PE-associated pathways, in addition to pTSS coverages of *CACNB2* and *NRF1* genes (Supplementary Figure S2). The clinical risk factors were maternal age, BMI, parity, past medical histories and method of conception. Similarly, the cfDNA profile, which was the ratio of pTSS coverages of *FLT3LG* and *EGF* genes (Supplementary Figure S3), and the same clinical risk factors as C_EPE_ were used to construct the late-onset PE classifier (C_LPE_).

**Figure 3:**
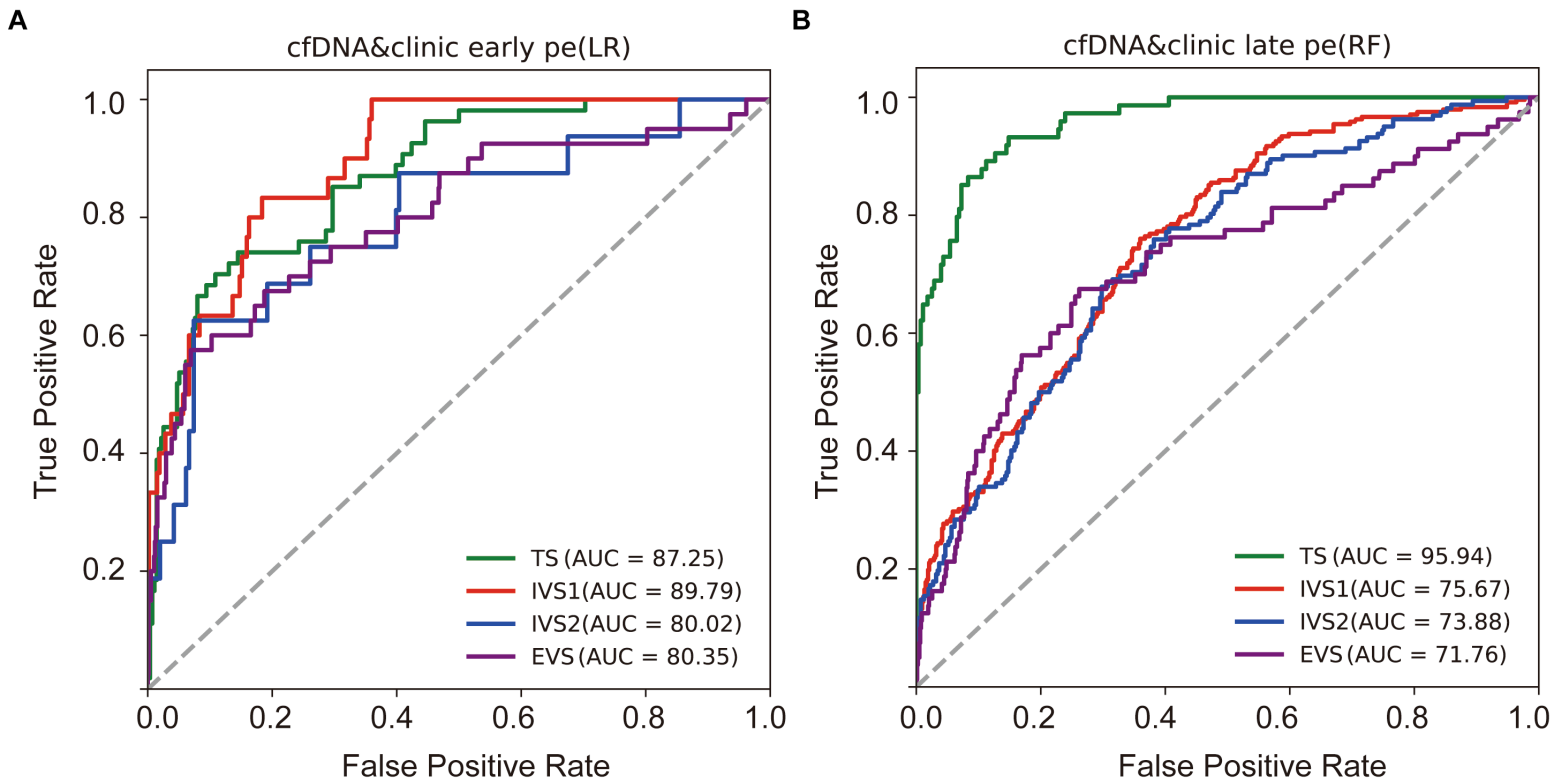
Performances of classifiers in predicting PE. Receiver operating characteristic (ROC) curves were drawn to evaluate performances of the early **(A)** and late-onset PE classifiers **(B)** based on the cfDNA profile and clinical risk factors in four datasets. TS, training dataset; IVS, internal validation dataset; EVS, external validation dataset.

In the training dataset, the C_EPE_ and C_LPE_ based on the logistic regression (LR) or random forest (RF) model achieved accuracies of 0.87 and 0.90, respectively (Table 2). The AUCs of C_EPE_ and C_LPE_ were 87 and 96% (Figures 3A,B). To further evaluate the accuracies of classifiers for predicting early- and late-onset PE, we validated C_EPE_ and C_LPE_ in two internal and one external validation datasets (Figure 1). The C_EPE_ exhibited AUCs ranging from 80 to 90% in three datasets (Figure 3A). For C_LPE_, the three validation datasets showed AUCs of 76, 74 and 72%, respectively (Figure 3B). With a false positive rate (FPR) of 10%, the average detection rates for predicting early- and late-onset PE were 63 and 48%, and the average PPVs were 33 and 55% in four datasets, respectively (Tables 2, 3).

**Table 2:** The performance of the logistic regression model for predicting early-onset PE in the training and three validation datasets.

	AUC (95% CI)	Acc	Sen	Spe	PPV	NPV
*Clinical risk factors*						
Training set	0.84 (0.79–0.84)	0.86	0.59	0.91	0.57	0.92
Internal validation dataset1	0.79 (0.75–0.80)	0.89	0.57	0.91	0.26	0.97
Internal validation dataset2	0.70 (0.65–0.72)	0.88	0.31	0.90	0.11	0.97
External validation dataset	0.79 (0.71–0.80)	0.89	0.58	0.90	0.23	0.98
*The cfDNA profile*						
Training set	0.69 (0.63–0.69)	0.80	0.30	0.90	0.37	0.87
Internal validation dataset1	0.79 (0.65–0.79)	0.87	0.27	0.91	0.14	0.96
Internal validation dataset2	0.76 (0.64–0.76)	0.89	0.38	0.91	0.14	0.97
External validation dataset	0.60 (0.54–0.62)	0.87	0.20	0.91	0.10	0.96
*Clinical risk factors + the cfDNA profile*						
Training set	0.87^NS, ***^ (0.81–0.87)	0.87	0.69	0.91	0.59	0.94
Internal validation dataset1	0.90^**, **^ (0.79–0.90)	0.89	0.63	0.90	0.27	0.98
Internal validation dataset2	0.80 ^NS, NS^ (0.72–0.81)	0.90	0.63	0.91	0.21	0.98
External validation dataset	0.80^NS, ***^ (0.72–0.82)	0.89	0.58	0.91	0.24	0.98

The data were presented in terms of area under curves (AUCs), accuracies, sensitivities, specificities, positive predictive values (PPVs), and negative predictive values (NPVs) of the final classifiers. The 95% confidence intervals of AUCs for the training and validation sets were computed based on 1,000 bootstrap samples. ^*^Represented the AUC of the classifier based on clinical risk factors and the cfDNA profile was greater than based on clinical risk factors or the cfDNA profile alone by DeLong’s test. *, ** and *** represented the different significance of *p* < 0.05, *p* < 0.01 and *p* < 0.001. Acc, accuracy; Sen, sensitivity; Spe, specificity; CI, confidence interval.

**Table 3:** The performance of the random forest model for predicting late-onset PE in the training and three validation datasets.

	AUC (95% CI)	Acc	Sen	Spe	PPV	NPV
*Clinical risk factors*						
Training set	0.96 (0.86–0.93)	0.90	0.88	0.90	0.71	0.97
Internal validation dataset1	0.74 (0.67–0.76)	0.72	0.31	0.90	0.59	0.74
Internal validation dataset2	0.72 (0.63–0.76)	0.73	0.28	0.91	0.55	0.76
External validation dataset	0.68 (0.61–0.71)	0.85	0.38	0.90	0.28	0.93
*The cfDNA profile*						
Training set	0.93 (0.71–0.81)	0.87	0.70	0.91	0.68	0.92
Internal validation dataset1	0.53 (0.45–0.55)	0.66	0.14	0.90	0.39	0.70
Internal validation dataset2	0.53 (0.45–0.56)	0.69	0.12	0.91	0.35	0.72
External validation dataset	0.50 (0.44–0.53)	0.84	0.10	0.92	0.11	0.91
*Clinical risk factors + the cfDNA profile*						
Training set	0.96^NS, *^ (0.87–0.94)	0.90	0.87	0.91	0.71	0.96
Internal validation dataset1	0.76^NS, ***^ (0.67–0.77)	0.73	0.33	0.91	0.63	0.75
Internal validation dataset2	0.74^NS, ***^ (0.64–0.77)	0.74	0.30	0.91	0.58	0.77
External validation dataset	0.72^*, ***^ (0.63–0.72)	0.85	0.40	0.90	0.29	0.94

The data were presented in terms of area under curves (AUCs), accuracies, sensitivities, specificities, positive predictive values (PPVs), and negative predictive values (NPVs) of the final classifiers. The 95% confidence intervals of AUCs for the training and validation sets were computed based on 1,000 bootstrap samples. ^*^Represented the AUC of the classifier based on clinical risk factors and the cfDNA profile was greater than based on clinical risk factors or the cfDNA profile alone by DeLong’s test. *, ** and *** represented the different significance of *p* < 0.05, *p* < 0.01 and *p* < 0.001. Acc, accuracy; Sen, sensitivity; Spe, specificity; CI, confidence interval.

In addition, classifiers based on the combination of the cfDNA profile and clinical risk factors showed higher AUC values in predicting early- or late-onset PE than based on clinical risk factors or the cfDNA profile alone in training and validation datasets (Tables 2, 3). Some of these reached statistical significance by DeLong’s test. These results showed the combination of the cfDNA profile and clinical risk factors enhanced performances of early- and late-onset PE prediction.

## 4 Discussion

In this study, we analyzed cfDNA coverages in gene promoter regions using NIPT data, namely pTSS coverage, and clinical risk factors from 2,727 pregnant women. We found that the combination of differential pTSS coverages and clinical risk factors could effectively distinguish PE patients from normal controls by machine learning methods. The early- and late-onset PE classifiers (C_EPE_ and C_LPE_) showed good performances for early- and late-onset PE prediction in four datasets (average AUCs of 0.84 and 0.80), with average accuracies of 89 and 80% and average detection rates of 63 and 48% at a 10% false positive rate. These results showed the C_EPE_ and C_LPE_ outperformed the FMF’s competing risk model in predicting PE for the Chinese (19).

Clinical risk factors in C_EPE_ and C_LPE_ included maternal age, BMI, parity, past medical histories and method of conception, exhibited significant differences in early- or late-onset PE samples compared to healthy controls. The relationship between these factors and the risk of PE had been previously evaluated (2, 12, 13). An effective method for disease prediction requires high accuracy, low cost, and easy application. While predicting PE using basic clinical information may be more accessible and cost-effective for clinicians, our investigation revealed variations in the performance of PE models based solely on clinical risk factors across different hospitals. The AUCs for early- and late-onset PE models based on clinical risk factors in three validation datasets ranged from 0.70 to 0.79 and from 0.68 to 0.74, respectively (Tables 2, 3). This shortcoming was mitigated by incorporating the cfDNA profile obtained through NIPT data. Although the addition of cfDNA profiles in external validation increased the AUC by only 1% compared to predictions based on clinical risk factors, the AUC for the early-onset PE classifier exceeded 0.79 in each hospital. Herein, we demonstrated NIPT data could be applied to predict pregnant women at high risk for PE. The predictive test that is a by-product of the NIPT would be inexpensive and could provide valuable supplementary clinical information (unrelated to aneuploidy screening). Even if its diagnostic performance is not excellent, it could assist clinicians in selecting pregnant women who need reassessment using additional techniques, especially for those without clinical risk factors. Therefore, our study has the potential to expand the value of NIPT for screening patients with PE.

While in some countries, NIPT is currently restricted to high-risk populations, the expanding utilization of NIPT data might have the potential to prompt initiatives in certain countries to provide financial support for NIPT through healthcare insurance or public health programs. This would ensure that a greater number of mothers can benefit from the advantages provided by NIPT. Moreover, as the cost of sequencing decreases in the future, the sequencing depth of NIPT continues to increase. We believe that the prediction effect of the NIPT data on PE will be further improved.

The cfDNA profile in C_EPE_ model contained eight genes, of which pTSS coverages of *FOSL2*, *CAMKK2*, *CCND1*, *ITPR1*, *PRKACB*, *WNT7B*, *CACNB2* and *NRF1* genes were stably differential between pregnant women with early-onset PE and healthy controls. *CAMKK2*, *CCND1*, *ITPR1*, *PRKACB* and *WNT7B* genes were enriched in more than two pathways associated with PE, including “Hedgehog signaling pathway” (35), “Hippo signaling pathway” (36), “AMPK signaling pathway” (37), “Apelin signaling pathway” (38), “Autophagy” (39), “Oxytocin signaling pathway” (40), “Wnt signaling pathway” (41) and “VEGFA-VEGFR2 signaling” (42). The AMPK signaling pathway has been repeatedly reported to be associated with PE based on omics data (37, 43). *FOSL2* is a transcription factor in the progression of angiogenesis. Down-regulated expression of *FOSL2* gene in PE placentas could cause placental vascular dysfunction, which is consistent with higher cfDNA coverage at its pTSS (44). The cfDNA profile in C_LPE_ contained pTSS coverages of *FLT3LG* and *EGF* genes. These two genes are enriched in “Ras signaling pathway,” “PI3K-Akt signaling pathway” and “MAPK signaling pathway” (45, 46). Of these, the PI3K-Akt and MAPK signaling pathways are critical for trophoblast function and implicated in underlying causes of preeclampsia, such as dysfunction of the placental endothelial nitric oxide synthase. The *EGF* family regulates the development of trophoblast and plays a role in trophoblast cell invasion. The *EGF* levels in plasma and serum have been verified to be significantly decreased (47, 48).

The strength of this study lies in the development and validation of PE classifiers within a larger population compared to other studies, albeit being a retrospective case–control study. The established early- and late-onset PE classifiers were validated in three datasets which means changes of these differential pTSS coverages and clinical risk factors are more common in patients with PE. Our study has some limitations. First, the C_EPE_ and C_LPE_ were constructed and validated from case–control cohorts of four hospitals. Although we retained the population differences between hospitals, the number of hospitals in this study was relatively small. It is necessary to validate these classifiers in a larger number of hospitals in the future. Second, this study is performed in China in specific maternal and environmental characteristics. It is imperative to validate these classifiers in different populations before advocating its use as a universal screening method. Third, NIPT data we used is single-end reads. It lacks information on the length of cfDNA fragments. A study verified that pregnant women with late-onset PE had a significantly higher cfDNA fragment size distribution compared to controls (25). The potential of combined this cfDNA signal with pTSS coverages for PE prediction needs to be further explored. Fourth, the accuracy of the C_LPE_ was lower than that of the C_EPE_. The reason might be that there is higher heterogeneity in the pathogenesis of late-onset PE. In the future, we hope to develop classification models for different subtypes of late-onset PE in larger samples.

## 5 Conclusion

The classifiers established by integrating cfDNA profiles could mitigate the performance variations observed in PE models based on clinical risk factors alone. This could potentially broaden the application of NIPT in PE screening in the future.

## Data availability statement

The original contributions presented in the study are publicly available. This data can be found in the CNGB Nucleotide Sequence Archive (CNSA: https://db.cngb.org/cnsa; access number: CNP0003877).

## Ethics statement

This study was approved by the Ethics Committees of Zhuhai Center for Maternal and Child Health Care, Shenzhen Baoan Women’s and Children’s Hospital (LLSC-2022-01-04-04-KS), Changsha Hospital for Maternal and Child Health Care (2022030) and Jiangmen Central Hospital ([2022]02) and BGI (BGI-IRB 22026). All participants provided written informed consent. The studies were conducted in accordance with the local legislation and institutional requirements. The participants provided their written informed consent to participate in this study.

## Author contributions

YY: Resources, Writing – review & editing. WX: Conceptualization, Formal analysis, Methodology, Validation, Visualization, Writing – original draft. SuZ: Resources, Writing – review & editing. SF: Resources, Writing – review & editing. FF: Conceptualization, Formal analysis, Validation, Writing – review & editing. JD: Writing – review & editing. XZ: Conceptualization, Formal analysis, Writing – review & editing. PT: Resources, Writing – review & editing. SW: Resources, Writing – review & editing. ZZ: Conceptualization, Writing – review & editing. WZ: Conceptualization, Writing – review & editing. LG: Project administration, Writing – review & editing. ZQ: Writing – review & editing. JiaZ: Project administration, Writing – review & editing. HP: Project administration, Writing – review & editing. JLin: Resources, Writing – review & editing. QuZ: Resources, Writing – review & editing. WC: Resources, Writing – review & editing. HuahL: Resources, Writing – review & editing. QiZ: Funding acquisition, Resources, Writing – review & editing. GX: Resources, Writing – review & editing. ZL: Resources, Writing – review & editing. ShZ: Resources, Writing – review & editing. CP: Resources, Writing – review & editing. ZX: Resources, Writing – review & editing. JinZ: Resources, Writing – review & editing. RZ: Resources, Writing – review & editing. XH: Resources, Writing – review & editing. HuaL: Funding acquisition, Resources, Supervision, Writing – review & editing. JLi: Conceptualization, Data curation, Investigation, Methodology, Supervision, Validation, Writing – review & editing. XR: Resources, Supervision, Writing – review & editing. LZ: Supervision, Writing – review & editing. JH: Resources, Supervision, Writing – review & editing.
