# Supplementary material for: Non-invasive prediction of preeclampsia using the maternal plasma cell-free DNA profile and clinical risk factors
Source: Front Med (Lausanne). 2024 Apr 17;11:1254467. doi: 10.3389/fmed.2024.1254467 (PMC11061442; doi:10.3389/fmed.2024.1254467)
Supplement: Supplementary file 1 [file Data_Sheet_1.docx]

Supplementary Material

**Supplementary Table 1.** Demographic and clinical characteristics of late-onset PE patients and healthy controls in the training and validation datasets

|  | Training set | | Internal validation dataset1 | | Internal validation dataset2 | | External validation dataset | |
| --- | --- | --- | --- | --- | --- | --- | --- | --- |
|  | Control | LPE | Control | LPE | Control | LPE | Control | LPE |
| Number of subjects | 276 | 74 | 528 | 242 | 406 | 162 | 779 | 80 |
| Maternal age (years) | 28.0 (26.0-31.0) | 32.0 (27.3-34.0)*** | 29.0 (27.0-31.0) | 29.0 (27.0-32.0)* | 31.0 (29.0-33.0) | 32.0 (29.0-34.0) | 29.0 (27.0-32.0) | 31.0 (28.0-35.0)** |
| Height (cm) | 160.0 (156.0-163.0) | 160.0 (155.0-164.0) | 160.0 (156.0-163.0) | 158.3 (155.0-162.0) | 161.0 (158.0-165.0) | 158.5 (156.0-162.0)*** | 160.0 (157.0-164.0) | 159.0 (155.0-161.3)* |
| Weight (kg) | 52.5 (47.0-57.0) | 56.0 (50.6-63.0)*** | 53.0 (48.0-58.0) | 55.8 (50.8-63.0)*** | 54.5 (50.0-59.0) | 58.0 (54.0-65.8)*** | 54.0 (50.0-60.0) | 58.7 (51.0-65.0)** |
| BMI (kg/m^2^) | 20.5 (18.8-22.5) | 22.0 (20.2-24.6)*** | 20.6 (19.1-22.7) | 22.1 (20.3-24.6)*** | 20.8 (19.6-22.4) | 23.0 (21.2-26.1)*** | 21.1 (19.5-22.9) | 23.2 (20.6-25.5)*** |
| GA at delivery (weeks) | 39.4 (38.6-40.1) | 37.5 (36.2-38.5)*** | 39.0 (38.0-40.0) | 38.6 (37.2-39.3)*** | 39.0 (39.0-40.0) | 39.0 (37.0-39.0)*** | 39.0 (38.0-40.0) | 38.0 (37.0-39.2)*** |
| Gravidity ≥ 1, n (%) | 276 (100.0) | 74 (100.0) | 528 (100.0) | 242 (100.0) | 406 (100.0) | 162 (100.0) | 779 (100.0) | 80 (100.0) |
| Parity ≥ 1, n (%) | 157 (56.9) | 25 (33.8)* | 298 (56.4) | 55 (22.7)*** | 164 (40.4) | 26 (16.0)*** | 279 (35.8) | 27 (33.8) |
| PMH ≥ 1, n (%) | 0 (0.0) | 2 (2.7)* | 0 (0.0) | 9 (3.7)*** | 0 (0.0) | 6 (3.7)*** | 0 (0.0) | 1 (1.3) |
| IVF, n (%) | 5 (1.8) | 21 (47.7)*** | 0 (0.0) | 30 (12.4)*** | 0 (0.0) | 9 (5.6)*** | 15 (1.9) | 10 (12.5)*** |

Data were shown as median values (interquartile range) of clinical characteristics or numbers (percentages) of samples. *, ** and *** represented the different significance of P<0.05, P<0.01 and P<0.001 between late-onset PE and control samples, respectively. Abbreviations: LPE, late-onset preeclampsia; BMI, body mass index; PMH, past medical history; GA, gestational age; IVF, in vitro fertilization.

**
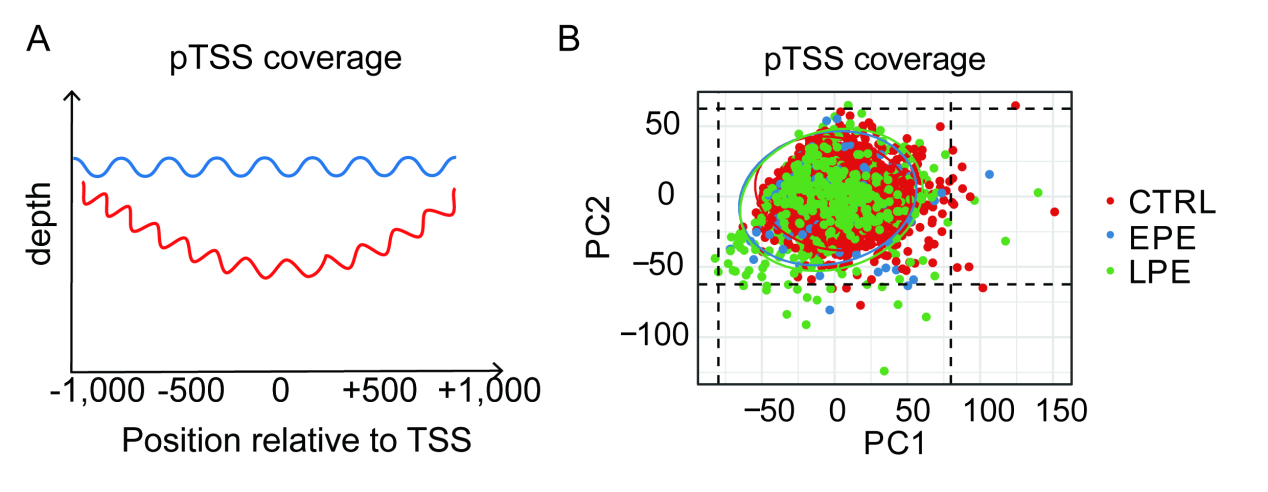
**

**Supplementary Figure 1.** The schematic diagram of the pTSS coverage. The normalized pTSS coverage at the pTSS calculated by using the reads per kilobase per million mapped reads (RPKM) method (A). Based on pTSS coverage results, the PCA excluded 3 early-onset PE, 22 late-onset PE and 15 control outlier samples from the training and validation datasets (B). CTRL: controls; EPE: early-onset PE; LPE: late-onset PE.


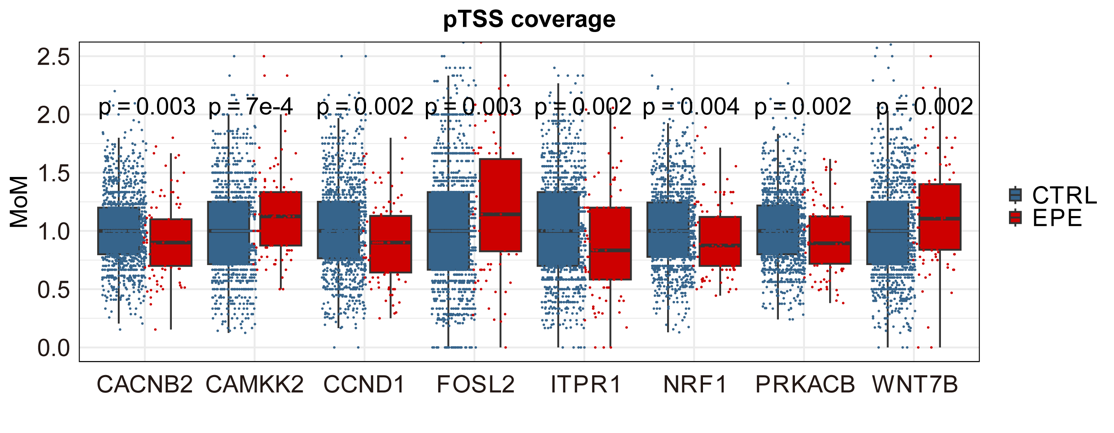


**Supplementary Figure 2.** The boxplots of pTSS coverages in early-onset PE and control samples. Boxplots showed the differences in pTSS coverges of *FOSL2*, *CAMKK2*, *CCND1*, *ITPR1*, *PRKACB*, *WNT7B*, *CACNB2* and *NRF1* genes between early-onset PE samples and controls in training and internal validation datasets. These pTSS coverages were used to construct the early-onset PE classifier. The Wilcoxon rank sum test was used to identify the significance of differences. EPE: early-onset PE; CTRL, controls.


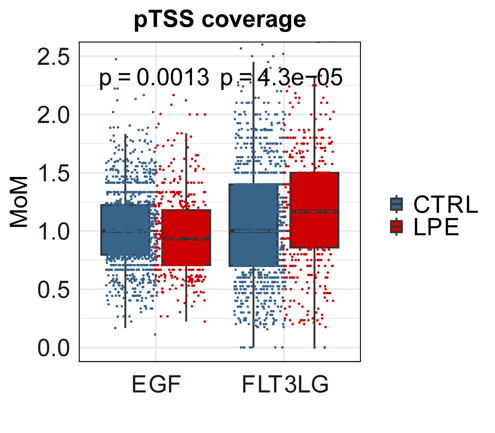


**Supplementary Figure 3.** The boxplots of pTSS coverages in late-onset PE and control samples. Boxplots showed the differences in pTSS coverges of *FLT3LG* and *EGF* genes between late-onset PE samples and controls in training and internal validation datasets. These pTSS coverages were used to construct the late-onset PE classifier. The Wilcoxon rank sum test was used to identify the significance of differences. LPE: late-onset PE; CTRL, controls.
